# Supplementary material for: Accuracy of Heart Rate Measurement Under Transient States: A Validation Study of Wearables for Real-Life Monitoring
Source: Sensors (Basel). 2025 Oct 13;25(20):6319. doi: 10.3390/s25206319 (PMC12568089; doi:10.3390/s25206319)
Supplement: Supplementary file 1 [file sensors-25-06319-s001.zip › File S1.pdf]

Data collection protocol: Overview datafile formats, firmware and software versions

Each device used in the study has different specifications and requires actions to obtain the recorded data. The complete dataset available from all devices was combined in Excel (CSV format). An overview of the firmware and software versions can be found in Table S1.

The 12-lead electrocardiogram (ECG) (CAM-14 module, GE Healthcare, Helsinki, Finland), recorded the electrical activity of the heart at 500 Hz. Since full disclosure ECG bands were not continuously accessible during the tests, the heart rate defined by the provided software CardioSoft (GE Healthcare, Helsinki, Finland) was used. This data was exported as an XML file and contained heart rate measurements every 5 or 10 seconds. This data was exported as an Excel file (CSV) under the export option 'time interval average' and contained breathing rate measurements every second. The Zephyr BioHarness 3.0 records electrical activity of the heart at 250 Hz using a single lead ECG. Based on the preceding 15 seconds of this signal, the heart rate is calculated and returned every second. The EmbracePlus (Empatica Inc., Cambridge, USA) records PPG signal at 64 Hz [47]. Based on this signal, the proprietary algorithms of Empatica calculate the heart rate and breathing rate every minute. These measurements are exported in an Excel file (CSV) and downloaded from the Empatica Cloud on AWS Cloud Services (Amazon Web Services Inc., Seattle, United States). The heart rate measurements of the consumer-grade devices were recorded via their propriety PPG technology and determined based on their propriety software. Raw PPG signals from the manufacturers are not properly accessible, nor are the specific sensor sampling rates or the specificities of heart rate calculations, such as the time window or number of peaks used. For the consumer-grade devices, the heart rate data was retrievable in the online platforms by recording an activity. When possible, the activity was exported through the online platform itself, as for the Garmin Vivosmart 4 (Garmin Ltd., Olathe, USA) via Garmin Connect and for the Withings Scanwatch (Withings, Issy-les-Moulineaux, France) via Withings Health Mate. If no direct export was possible, the activity was transferred from the online platform to Strava (Strava Inc., San Fransisco, USA) in order to facilitate export. The Strava connection for the Fitbit Sense 2 and Fitbit Charge 5 (Google LLC, Mountain View, USA) from the Fitbit Health and Fitness Platform was made via FitToStrava [48]. The Strava connection for the WHOOP 4.0 (WHOOP, Boston, USA) from the WHOOP platform was made directly. The exported activity was either in Excel, FIT or TCX format. The FIT format was converted to Excel using FIT File Viewer [49] and the TCX format was converted using the XML data input function of Excel.

Note that additional pre-processing was conducted for the Withings Scanwatch. Its export file format contained three columns: 'Start time', 'Duration' and 'HR'. The interpretation of this format is explained as 'a duration of [X,Y,Z] with times of [A,B,C] seconds mean a heart rate of X for A seconds, then a heart rate of Y for B seconds, and then a heart rate of Z for C seconds' [50]. Inconsistencies in this description were found. For example, the total elapsed time between the first and the last starting hour from the column 'Start time' plus the duration of the last measurement period was different from the total duration provided in 'Duration' column. Since no additional information was provided by Withings, the time stamps in the column 'Start time' were used and the corresponding HR value was calculated as the average of the values between the brackets in the column 'HR'. This average was used as the measured HR of the Withings Scanwatch.

Table S1: Overview of the available data in the export files and the format of these files for the different devices.

Device	Version (firmware and software)	Datafile format
12-lead ECG	Firmware CAM-14 module; software GE CardioSoft V6.73	.xml

Zephyr Bioharnas 3.0	Firmware RF module v.3.208; boot software v.1.3.1.0; app software v.1.7.8.0 Log Downloader 9500.0078.V1c (1.0.38.0)	.xlsx
EmbracePlus	Mobile App version 5.6.9 (24031413), software version 7.4.1	.xlsx
Garmin Vivosmart 4	App version 4.64.1.2; software automatically updated (Garmin Support Center, 2023)	.fit
Fitbit Charge 5	App version was 3.76; software version 20001.188.58	.tcx
Fitbit Sense 2	App version 3.76; software version 128.6.17	.tcx
Withings Scanwatch	Software version 2421	.xlsx
WHOOP 4.0	Firmware 41.11.7.0; bluetooth firmware 17.2.2.0; App version 4.6.170	.fit

Dataset descriptions

Frequencies at which data is provided is variable, ranging from 1 to 30 seconds. Hence, to conduct the statistical analyses for validation and assess the impact of smoothing on performance, the collected data was combined into subsets for various frequencies. Generation of subsets was done by first aligning the signals from the two concerned devices (i.e., the device and the reference device) based on the start time of the test, utilizing the timestamps provided by the respective manufacturer. Subsequently, observations were paired at a specific frequency (i.e., the frequency chosen for validation). If the frequency for validation was lower than the frequency at which the device provided data originally, the data was either averaged over a time frame (e.g. 10 or 60 seconds) or data was selected at the specific timestamp (per second) to match the chosen frequency for analysis. If datapoints were missing in the time frame for averaging (e.g. 10 or 60 seconds) or at the specific timestamp (per second), pairwise deletion was conducted and this pair was not included in the concerning dataset. An overview of the generated datasets can be found in Table S2.

- **Datasets against 12-lead ECG**

The validation of the heart rate measurements against the gold standard reference (12-lead ECG CardioSoft, GE Healthcare) is conducted at three selected frequencies since heart rate by the ECG was provided every 5 to 10 seconds. For each frequency a subset of observations is included from the originally collected data and paired to the corresponding ECG observations. In the “Per second” subset, data from the devices is selected per second at ECG frequency. This allows validation against ECG is at the lowest possible frequency. In the “10-second” subset, data from the devices is averaged over a window of 10 seconds previous to ECG frequency. Assuming the ECG uses a window of previous data to calculate the heart rate, this allows validation against ECG for which it is most probable that both the devices and ECG used similar data for their heart rate calculations. In the “60-second” subset, data from the devices and the ECG is averaged over a window of 60 seconds. Since the largest frequency at which heart rate data is provided (i.e. 60 seconds by the EmbracePlus), this allows validation against ECG so that performance can be systematically compared amongst devices. An illustrative example of the generation of the per-second, 10-second, and 60-second datasets for the Fitbit Charge 5 in comparison with the 12-lead ECG is provided in Figure 1.

- **Dataset against Zephyr Bioharness 3.0**

To validate the EmbracePlus and consumer-grade wearables at the highest frequency possible (i.e. the frequency at which the relevant device provided data), the devices were validated against a highly accurate reference device which provided measurements every second (i.e. Zephyr Bioharness 3.0).

This data subset is generated by pairing the observations from both devices (i.e. the Zephyr and the relevant device) per second at the wearable frequency.

Table S2: Overview of generated datasets for heart rate to conduct validation analysis.

Reference	Wearables (to validate)	Data Alignment Method	Aggregation Level of Resulting Dataset
12-lead ECG	Zephyr Bioharness 3.0, EmbracePlus, Fitbit Charge 5, Fitbit Sense 2, Garmin Vivosmart 4, WHOOP 4.0, Withings Scanwatch	At timestamp ECG, average 10 seconds before	10 second averages
12-lead ECG	Zephyr Bioharness 3.0, EmbracePlus, Fitbit Charge 5, Fitbit Sense 2, Garmin Vivosmart 4, WHOOP 4.0, Withings Scanwatch	Take 60 second averages	60 second averages
12-lead ECG	Zephyr Bioharness 3.0, EmbracePlus, Fitbit Charge 5, Fitbit Sense 2, Garmin Vivosmart 4, WHOOP 4.0, Withings Scanwatch	At timestamp ECG, match per second	Per second
Zephyr Bioharness 3.0	EmbracePlus, Fitbit Charge 5, Fitbit Sense 2, Garmin Vivosmart 4, WHOOP 4.0, Withings Scanwatch	At timestamp wearable, match per second	Per second

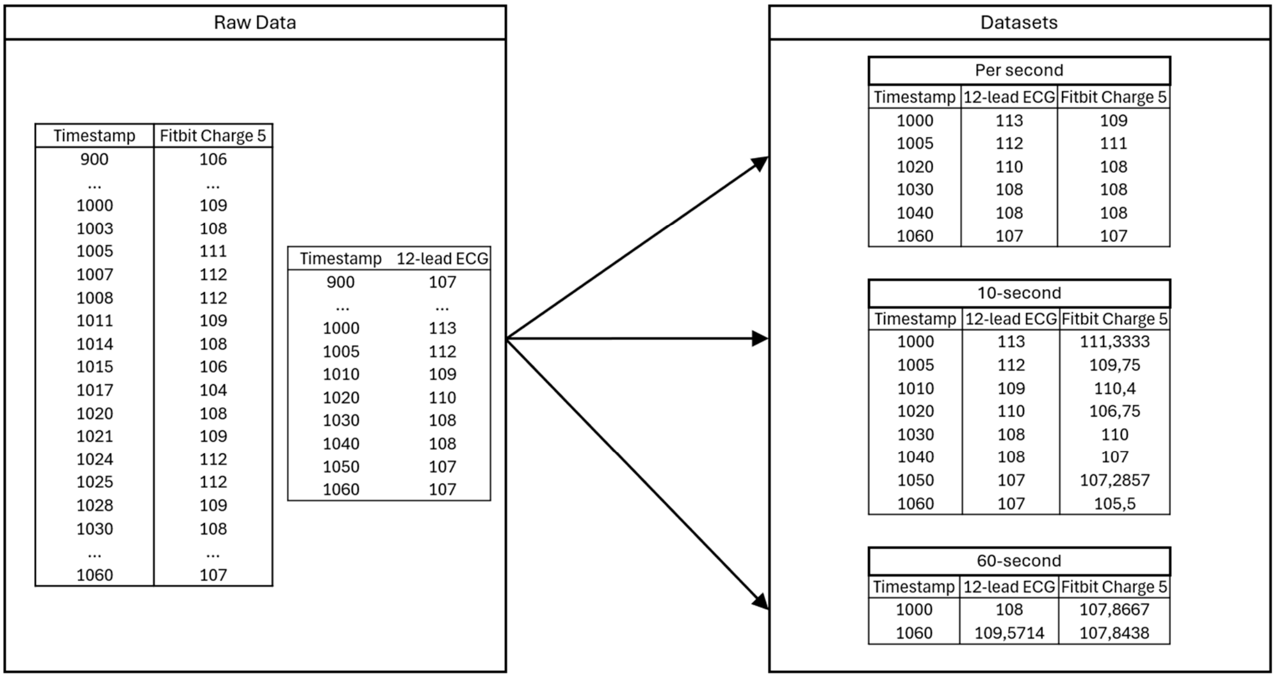

Figure 1: Illustrative example of per second, 10-second, and 60-second dataset construction for Fitbit Charge 5 validation against the 12-lead ECG.

<u>Encountered issues in data collection</u>

The EmbracePlus and the consumer grade wearables did not record heart rate data for the first four participants due to unavailability of the wearables on the first day of testing. In total, all wearables were worn by 20 participants, but for some participants the recording of the consumer grade wearables failed due to a variety of issues. For five participants, the same account was used to collect data of both Fitbits. After visual inspection, it seemed that the data of both recordings was merged into one identical heart rate series. The merged data was excluded for validation and for the other participants two different

accounts were used. Four recordings of the Garmin Vivosmart 4 were not saved correctly due to problems with starting the activity and accidentally touching the wrong icon by the researcher. In total 11 recordings of the WHOOP 4.0 failed due to either not establishing the Strava connection, or not having the Bluetooth connection with the smartphone to start an exercise measurement (this can only be done through the app and not via the wearable itself). The recordings of the EmbracePlus were successful for heart rate, however in the breathing rate measurements errors such as 'device_not_worn_correctly' and 'worn_during_motion' often occurred, limiting this breathing data available for analysis.
